# Patient Involvement in the Health Technology Assessment Process in Taiwan

**DOI:** 10.3389/fmedt.2021.732160

**Published:** 2022-01-21

**Authors:** Kuei An Chen, Li Ying Huang, Churn Shiouh Gau

**Affiliations:** ^1^Division of Health Technology Assessment, Center for Drug Evaluation, Taipei, Taiwan; ^2^School of Pharmacy, National Taiwan University, Taipei, Taiwan

**Keywords:** patient involvement, health technology assessment, Taiwan, NHIA, Pharmaceutical Benefit and Reimbursement Scheme (PBRS)

## Abstract

The COVID-19 pandemic initially had a smaller impact on Taiwan than on most other industrialized countries. However, an outbreak in late April 2021 led to a sharp surge in cases from mid-May 2021. Patient involvement in the health technology assessment (HTA) process, however, was not much affected by this; virtual meetings were implemented. This descriptive paper presents an overview of patient involvement in the HTA process in Taiwan via the National Health Insurance Administration (NHIA) online submission platform, participation in appraisal committees, education programs, and cooperation with patients' organizations, and outlines its progress and challenges. The National Health Insurance Act, amended in 2013, protects patients' rights and invites them to voice their opinions, which are then presented to the relevant authority. Based on this act, various mechanisms have been developed to involve patients, caregivers, and patient organizations in both the HTA and the reimbursement process. Prior to the Pharmaceutical Benefit and Reimbursement Scheme (PBRS) Joint Committee meeting, the NHIA built an online platform that allows patients to submit their opinions, which are then incorporated into the HTA reports. The results are also discussed with patient representatives, following which the related documents are published on the NHIA website. From May 2015 to December 2020, 30 patients' insights were published before the PBRS Joint Committee meetings. Of these, 19 (63%) were related to oncology cases. In Taiwan, approaches to fostering patient engagement include the use of a platform for patients' and patients groups' input, among others. Although patient engagement is important for understanding the needs of the target patient population, challenges in ensuring timely patient engagement and provision of relevant resources remain. In addition, further efforts are needed to implement and improve the visibility of patient input in the HTA process.

## Introduction

In 1995, the single-payer National Health Insurance (NHI) program was established in Taiwan. This mandatory social health insurance is internationally known for its low premiums and co-payments. The NHI covers more than 99% of Taiwan's population (1). Taiwan began conducting health technology assessments (HTAs) in 2007 to support the National Health Insurance Administration's (NHIA) reimbursement policies on new drugs (2). Adhering to medical ethics, the HTAs consider the health and well-being of all citizens as well as the cost-effectiveness of new medical technology within the financial framework of the NHI program. The HTA department operates under the supervision of the Center for Drug Evaluation (CDE) (2). In 2013, Taiwan implemented the second-generation National Health Insurance Act 2, with the HTA process, the composition of the Pharmaceutical Benefits and Reimbursement Scheme (PBRS) Committee, and transparency being written into the law. In addition, patient groups could now be invited to participate in PBRS Committee meetings (3).

The COVID-19 pandemic initially had a smaller impact on Taiwan than on most other industrialized countries (4, 5). However, an outbreak in late April 2021 led to a sharp surge in cases from mid-May 2021, mainly affecting the Greater Taipei area (6). Patient involvement in the HTA process, however, was not much affected by this; virtual meetings were implemented. In this paper, we focus on the development of patient involvement activities in Taiwan, while also comparing the current situation with that prior to the pandemic.

### Patient Involvement in HTA Mechanism

In 2015, the NHIA announced the launch of a new page on its website specifically allowing patients, caregivers, and patient groups to submit their opinions about new drugs or medical devices. Thirty days before the scheduled PBRS Committee meeting, all input from the online platform was collected and summarized by the CDE/HTA division. The findings were then sent to the PBRS Committee meeting for consideration (7). The online platform was designed to include four main domains that would accept information regarding new treatment as well as personal details, the Declaration of Interest statement, and a statement from the patients regarding their perspectives on the experience (8). In 2016, the NHIA published a patient involvement guideline to assist the patient/caregiver/patient groups in expressing their opinions on the online platform more efficiently (7). Based on this guideline, only opinions on new technology that meet certain criteria are currently collected. For new drug applications, patient opinions are collected only if the product being discussed is related to treating the diseases included in the NHI's major illnesses/injuries list (7). On the online platform, patients, caregivers, and patient organizations can share seven kinds of information: the method of information gathering; experiences of living with the conditions/diseases; experiences of the traditional and new treatments; expectations regarding the new treatments; effects on caregivers with/without the new treatments; and other opinions (8). Patients' opinions are collected for at least 30 days before the application is listed on the agenda for the PBRS Joint Committee meeting (7, 9). The platform's questionnaire includes the following seven questions (8):

- How do you gather opinions? (personal experience, website, interview, focus group, survey, or others)
- How does your disease or condition affect your or your family's daily life?
- If you have not used this new treatment before, what is your current treatment? How effective is it? Have you encountered any adverse reactions or uncontrollable situations?
- If you have not used this new treatment before, what are your expectations from it? What kinds of conditions, adverse reactions, or quality of life do you hope for?
- If you have used this new medication before, how effective is it? Are there any adverse reactions? How does it affect your or your family's daily life?
- If you are a caregiver, please describe what kinds of conditions or adverse reactions on the part of the patient have affected your daily life.
- Is there anything else specifically related to your disease or treatment that you would like to mention?

The CDE/HTA team retrieves all opinions received via the platform, summarizes them, and then incorporates them into the HTA report. The report is published before the PBRS Joint Committee meeting, allowing stakeholders to learn about patients' experiences (7).

Although the webpage is established, the questions are simple and cannot adequately solicit information about patients' unmet medical needs. A participant may not know whether they need to answer all the questions or only a few. Thus, the current method is quite primitive, and changes must be made so that patients' voices can be heard clearly. Between 2019 and 2020, the CDE/HTA team set up more practical guidelines to help patients get their voices heard. It is hoped that these guidelines fulfill their purpose and motivate patients and patient groups alike.

Patients can participate in a PBRS Joint Committee meeting in two ways. First, two patient representatives are invited to attend the PBRS Joint Committee meeting (7, 9) and second, in a resubmission case, the NHIA can invite two disease-specific patient representatives to voice their opinions during the meeting (7, 9). In 2019, the NHIA revised the regulations governing the joint establishment of the NHI drug-dispensing items and fee schedule to allow two patient representatives to participate in the PBRS Joint Committee meeting routinely (9). The CDE subsequently developed a project to assist patient representatives in understanding more about the HTA process, diseases, and patient voices. The CDE/HTA team also holds a pre-meeting for patient representatives, beneficiary representatives (consumers) and case-related patient organizations, who have provided input on the platform to discuss patients' perspectives before the PBRS Joint Committee meeting. Moreover, in a resubmission case, the NHIA can invite patient organization representatives and listen to their opinions in the PBRS Joint Committee meeting for 10 min (7). Since 2016, the PBRS joint meeting has invited patient organization representatives to state their opinions.

### Cooperation With Patients' Organization

In March, 2016, the Taiwan Alliance of Patients' Organization (TAPO) was established. Since then, more than 18 patient groups have joined the organization. All of which have an equal right to voice their opinions. The TAPO has also joined the International Alliance of Patients' Organization (IAPO) (8). The CDE, together with other related agencies and various patient groups, prepares HTA reports and interacts with patients to ensure a better understanding of the HTA process and an effective, transparent government policy. From May 2015 to December 2020, 30 patient inputs were published before the PBRS Joint Committee meetings. Of these, 19 (63%) were related to oncology cases.

In some technology assessment projects, the CDE/HTA conducted interviews with patients regarding their experiences with trans-oral robotic surgery—four via telephone and one face-to-face. In this case, patient organizations assisted the CDE/HTA in finding appropriate patients to ensure that the final report included the views of those who had had experiences in open surgery, chemotherapy, or radiation therapy, so that these could be referenced by decision-makers.

In addition, there were some other projects related to the improvement of the patient involvement mechanism that involved a high degree of cooperation with patient organizations. In these projects, the CDE/HTA not only reviewed the experiences of patients from other countries, but also surveyed more than 10 patient organizations. It then set up an advisory committee with experts—which included patient representatives of the PBRS—and conducted six interviews with patient organizations. Through such cooperation, the CDE/HTA hoped that the patient involvement mechanism could become more structured and adaptable to local conditions.

### Education of Patient Advocacy Groups

In 2016, the CDE/HTA established a patient involvement taskforce and initiated a series of educational programs for patients, caregivers, volunteers in hospitals, and patient organizations. Its main purpose was to introduce HTAs, the reimbursement process, and patient involvement mechanisms in Taiwanese populations. Since then, the CDE/HTA has conducted more than 15 training courses for patients, caregivers, hospital volunteers, and patient organizations focused on various disease types, like systemic lupus erythematosus, rheumatoid arthritis, cancer, psoriasis, development disability, and end-stage renal disease. These training courses were held across Taiwan, from Taipei to Kaohsiung, and even on the island of Penghu. More than 300 people took part. In addition, two international conferences for stakeholders were hosted in Taiwan, focusing on the questions “What is HTA?” and “How do we include patient voices in evidence?”

In 2018, the CDE/HTA developed instructions for patients, caregivers, and patient organizations using the online platform. In the following year, a review of patient involvement in HTA across various countries was prepared by decision-makers. This was meant to serve as a reference and provide information to patient organizations regarding patient involvement procedures in various countries.

In summary, Table 1 shows the mapping of patient involvement in the health technology assessment process in Taiwan.

**Table 1:** The mapping of patient involvement in the health technology assessment process in Taiwan.

**Year**	**Key progress**
1995	NHI program established
2007	Began conducting HTAs
2013	PBRS established, invite patient input
2015	Webpage/online platform established for patient input−4 kinds of information
2016	Patient involvement guideline on use of online platform
2016	Patient organizations invited to present at PBRS Joint Committee meeting
2018	Instructions on using online platform
2019	Two patient representatives on PBRS Joint Committee meeting
2020	Online platform established for patient input—extended to seven kinds of information for HTA report (released before PBRS meeting)
2020	Through the pre-meeting mechanism, discussion on patients' perspectives is conducted before the PBRS Joint Committee meetings, and feedback provided to them acts as input for the online platform
2020–2021	Patient opinions are put in HTA reports

## Discussion

In Taiwan, patients participating in HTA and the reimbursement decision-making process are fully supported by the NHIA. In this process, the CDE/HTA team plays a crucial role in supporting not only the NHIA, but also patients and patient organizations. Since 2015, patients have been able to engage in both processes in Taiwan through various mechanisms. Prior to the PBRS Joint Committee meetings, patients can report their experiences through the online platform; the CDE/HTA then summarizes these experiences and incorporates them into the HTA report. Discussions with the relevant stakeholders are also conducted before the meeting. Two patient representatives can participate in the meeting along with representatives of disease-specific patient organizations. After the PBRS meetings, the meeting documents and audio recordings are published on the NHIA website and made fully available to stakeholders and citizens. The deliberative process is thus more transparent and interdisciplinary.

Other HTA bodies, like the National Institute for Health and Care Excellence (NICE) in England (10), The Canadian Agency for Drugs and Technologies in Health (CADTH) in Canada (11), and Scottish Medicines Consortium (SMC) in Scottish (12), have formal templates they use to collect patient evidence from patient organizations. The Pharmaceutical Benefits Advisory Committee (PBAC) in Australia has also constructed an online platform for consumers to provide their opinions (13). Taiwan's patient involvement process is similar to the PBAC's.

The importance of the patient perspective in HTA is increasingly appreciated.

However, some challenges remain. First, despite the multiple mechanisms that allow patients to engage with the HTA and the reimbursement process, the impact of such decision-making remains unclear. Few patients have chosen to share their experiences, especially those involving medical devices, via the online platform. This is likely because many patient organizations still are not aware of the platform, even though it is a major facilitator of patient involvement. Second, both the HTA agency (14) and patient organizations lack human resources. The agenda for each PBRS Joint Committee meeting is published approximately seven days in advance (9), and patient representatives are expected to prepare patients' opinions on every single product in this duration. The scheduling leaves them with little time to get to know each case.

Because of this limitation, the CDE/HTA team references the guidelines The Health Technology Assessment international (HTAi) Interest Group for Patient and Citizen Involvement in HTA (PCIG) project has developed for patient organizations (15). Based on the NHIA's support, the questions on the platform are modified to cover different domains. Adopting the CDE/HTA team's suggestions has made the platform more comprehensive.

## Conclusion

Patient involvement in the HTA process in Taiwan has shown that results can be delivered even when resources are significantly more limited than those in many Western countries. Taiwan's policy serves as a model for middle-income countries seeking to build patient involvement in the HTA framework. As HTA is interdisciplinary (16), it is important to obtain views on patients' involvement in HTA from people worldwide (15). Taiwan began involving patients in the HTA decision-making process in 2015. The practice is new, and the process still requires adjustments and modifications based on the experiences gained over time. Patient involvement is encouraged through the use of a patient input platform, group conversations, and other methods. Although patient participation is essential for understanding the needs of the target population, challenges concerning timely involvement and resources remain. Further efforts are needed to implement and enhance the visibility of patient input in the deliberative process.

## Data Availability Statement

The original contributions presented in the study are included in the article/supplementary material, further inquiries can be directed to the corresponding authors.

## Author Contributions

KC, LH, and CG contributed to the design and implementation of the research, to the analysis of the results, and to the writing of the manuscript. All authors contributed to the article and approved the submitted version.

## Funding

The authors thank the National Health Insurance Administration (NHIA), Ministry of Health and Welfare (MOHW) for their financial support.

## Conflict of Interest

The authors declare that the research was conducted in the absence of any commercial or financial relationships that could be construed as a potential conflict of interest.

## Publisher's Note

All claims expressed in this article are solely those of the authors and do not necessarily represent those of their affiliated organizations, or those of the publisher, the editors and the reviewers. Any product that may be evaluated in this article, or claim that may be made by its manufacturer, is not guaranteed or endorsed by the publisher.
